# *In Vivo* Open-Bore MRI Reveals Region- and Sub-Arc-Specific Lengthening of the Unloaded Human Posterior Cruciate Ligament

**DOI:** 10.1371/journal.pone.0048714

**Published:** 2012-11-07

**Authors:** Alison J. King, Qunli Deng, Randy Tyson, Jonathan C. Sharp, Jarod Matwiy, Boguslaw Tomanek, Jeff F. Dunn

**Affiliations:** 1 Department of Radiology, Faculty of Medicine, University of Calgary, Calgary, Alberta, Canada; 2 McCaig Institute for Bone and Joint Health, University of Calgary, Calgary, Alberta, Canada; 3 Institute for Biodiagnostics – West, National Research Council of Canada, Calgary, Alberta, Canada; 4 Institute for Biodiagnostics, National Research Council of Canada, Winnipeg, Manitoba, Canada; Northwestern University Feinberg School of Medicine, United States of America

## Abstract

Open-bore MRI scanners allow joint soft tissue to be imaged over a large, uninterrupted range of flexion. Using an open-bore scanner, 3D para-sagittal images of the posterior cruciate ligament (PCL) were collected from seven healthy subjects in unloaded, recumbent knee extension and flexion. PCL length was measured from one 2D MRI slice partition per flexion angle, per subject. The anterior surface of the PCL lengthened significantly between extension and flexion (*p*<0.001). Conversely, the posterior surface did not. Changes were not due to the PCL moving relative to the 2D slice partition; measurements made from 3D reconstructions, which compensated for PCL movement, did not differ significantly from measurements made from 2D slice partitions. In a second experiment, videos of knee flexion were made by imaging two subjects at several flexion angles. Videos allowed soft tissue tracking; examples are included. In a third experiment, unloaded knees of seven healthy, recumbent subjects were imaged at extension and at 40°, 70°, 90°, 100°, 110° and 120° flexion. The distance between PCL attachments increased between extension and 100°, and then decreased (*p*<0.001). The anterior surface of the PCL lengthened over the flexion angles measured (*p*<0.01). The posterior surface of the PCL lengthened between extension and 40° and then shortened (*p*<0.001). Both attachment separation and anterior surface length increased dramatically between extension and 40°, but varied less afterwards. Results indicate that PCL dynamics differ between terminal extension and active function sub-arcs. Also, attachment separation cannot predict the lengthening of all parts of the PCL, nor can lengthening of one part of the PCL predict the lengthening of another part. A potential connection between lengthening and loading is discussed. We conclude that low-field MRI can assess ligament lengthening during flexion, and that the dynamics of the PCL for any given region and sub-arc should be measured directly.

## Introduction

The majority of patients with ruptured posterior cruciate ligaments (PCL) have enough knee stability to return to activity without surgical PCL reconstruction [1]. This suggests that the PCL plays only a minor role in knee stability. However, recent studies have demonstrated that chronically PCL-deficient knees are at higher risk of developing degenerative changes than are intact knees [1], [2], [3], [4]. This shows that the PCL is important for long-term knee health. Unfortunately, the contribution that the PCL makes to knee stability remains poorly understood. An improved understanding of the *in vivo* function of the PCL will facilitate improvements in operative and non-operative therapeutic interventions for PCL-deficiency [5].

Magnetic Resonance Imaging (MRI) is ideal for studying the soft tissues of a joint, such as the PCL, because soft tissues appear clearly in MR images, and imaging is not blocked by bone. However, clinical, closed-bore MRI scanners do not permit imaging of the knee between about 45° [6], [7], [8], [9] and 140° flexion [10] because the leg becomes restricted by the wall of the scanner. This is a significant limitation because the flexion sub-arc over which most daily activities occur spans from a lower limit of 10° or 30° flexion to an upper limit of 120° [11], [12]. This is called the “active function” sub-arc. Although one laboratory has succeeded in imaging knees in up to 90° flexion in a closed-bore scanner [13], this still leaves up to 44% of the active function sub-arc uninvestigated. Kinematics from the “terminal extension” sub-arc (extension to an upper limit of 10–30° flexion) should not be extrapolated to the active function sub-arc because each sub-arc has different bone kinematics including that each sub-arc rotates around a different facet centre [11], [12]. The effect of differing bone kinematics on soft tissue deformation has not been established.

Open-bore MRI scanners allow knees to be imaged over all of the terminal extension and active function sub-arcs. A modest number of studies on soft tissue deformation during flexion have been conducted *in vivo* on such open-bore MRI scanners [14], [15], [16], [17]. Even when using an open-bore scanner, studying PCL deformation is complicated. The tibia twists and slides relative to the femur [18], causing the PCL to follow a different path relative to the femur after knee flexion. These changes cannot be captured in a single imaging plane.

This paper describes three experiments that examine the deformation of the PCL during knee flexion. In Experiment 1, we hypothesized that moving the knee from extension to deep flexion in an unloaded open kinetic chain motion (i.e. flexion of only the knee when the foot is not fixed, but free to move) would cause gross changes in PCL length, as measured from a single imaging plane. To ensure that these differences were not caused by the PCL moving relative to the single imaging plane, measurements were compared to those taken from 3D reconstructions of the PCL, which could follow the new path of the PCL after joint flexion. In Experiment 2, we verified that we could make stop action videos of the PCL between knee extension and flexion. To test observations made from the videos, we performed Experiment 3. In Experiment 3, we hypothesized that PCL shape change was sub-arc-specific and region-specific. To test this, unloaded knees were imaged at several flexion angles between extension and flexion, and the PCL was measured. It was concluded that PCL shape change is sub-arc specific and region-specific. Therefore PCL shape change should be measured directly rather than being inferred by other measures.

## Methods

### Ethics Statement

The protocol was approved by the University of Calgary’s Conjoint Health Research Ethics Board. All subjects had the relevant Experiment explained to them orally and in writing. All subjects gave written consent before participating in the study.

### Scanner, Radio Frequency Coil and Sequences

**Figure 1:**
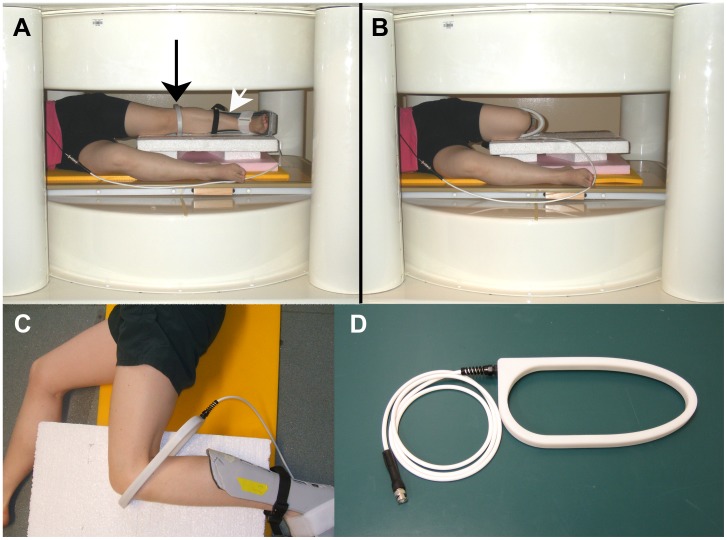
The open-bore MRI scanner and the radio frequency (RF) coil. A subject lying on her side in the scanner wearing the RF coil (black arrow) and foot brace (white arrow), with her knee in relaxed extension (A). Same subject with her knee in flexion (B). An aerial view of the RF coil around a flexed knee (C). The RF coil (D).

Experiments were conducted on a recumbent, 0.2T, permanent magnet, four-post, open-bore MRI scanner with a 45 cm gap between upper and lower pole plate covers (Cirrus, MRI-Tech, Canada) (Figure 1A and 1B). The console was custom-built (TMX, National Research Council of Canada, Institute for Biodiagnostics, Manitoba, Canada) [19].

A custom-made, single-turn radio frequency (RF) coil was used. It had an open geometry that allowed the knee to flex between extension and ∼120° (Figure 1C). The RF coil had a capacitively-coupled, transmit and receive design, with an oval-shaped internal aperture of 110 mm on the short axis and 260 mm on the long axis. It was 20 mm wide and 13 mm deep (Figure 1D). The RF coil provided a signal-to-noise ratio (SNR) of 40 and 29 for the sequences described in Table 1 for Experiments 1 and 3 respectively. SNR was calculated using the following equation [20]:

SNR = (0.655xmean voxel signal)/standard deviation of the signal-free background.

**Table 1:** MR Imaging parameters.

Parameter	Experiment 1	Experiment 2	Experiment 3
Pulse sequence	3D SSFP-FID	2D SSFP-FID	3D SSFP-FID
Flip angle (°)	80	80	120
TE (ms)	10	10	6.8
TR (ms)	20	20	14.3
Matrix	128×128×32	192×192	128×128×32
Field of view (mm)	180×180×96	180×180	180×180×96
Voxel size (mm)	1.4×1.4×3	0.94×0.94×5	1.4×1.4×3
Bandwidth (kHz)	80	20	20
Averages	4	4	2
Total imaging time	5min32s	19.4s	1min58s

A 2D sequence was used to acquire images for the 2D video in Experiment 2. A 3D sequence was used for all other scans. 3D acquisitions from Experiments 1 and 2 were reconstructed in 3D during post-processing. The 3D sequence ensured that the coordinate system was the same for each para-sagittal partition of a given scan. 3D sequences were used also because of their theoretical improvement in SNR per unit time over 2D versions of the same sequence [21]. A Steady State Free Precession-Free Induction Decay (SSFP-FID) pulse sequence [22] was used. During iterative optimization of the 3D SSFP-FID sequence, a flip angle of 80° or greater provided a better SNR than flip angles of 60° or 40°. Slice thicknesses of 1 mm resulted in an SNR of 8 (quite low), and therefore a slice thickness of 3 mm was used. A slice thickness of 3 mm is consistent with values used on clinical MRI scanners. The parameters selected for the three different Experiments described below are summarized in Table 1.

### PCL Imaging Procedures Common to all Experiments

Subjects recruited into the study had no history of knee injury, pain or disease in their left knees. Subjects lay on their right side in the scanner with a level platform supporting their left leg at about hip height. A rigid foot brace, which extended from the bottom of the foot to mid-shank, held the left foot at 90° flexion, with minimal internal or external rotation relative to the shank (Figure 1A). The brace standardized foot position between flexion angles and subjects, and supported the subject’s foot during imaging, reducing fatigue. Besides brace and body weight, the knees were unloaded. The subject actively extended his or her knee, and then relaxed it onto the supports. The knee’s flexion angle was measured. Knees were not at 0° during “extension” because they tended to flex once relaxed onto the supports. The flexion angle at “extension” was subject-specific and depended on the subject’s flexibility and how well the supports fitted the subject’s body. This flexion angle is called “relaxed extension” in this paper.

The subject’s knee was centered in the magnet and para-sagittal images were acquired along the PCL. After imaging the knee at extension, the subject’s thigh was held stationary while the knee was gently flexed to the Experiment-specific flexion angles described below. The open bore of the scanner allowed the subject’s joint to be moved from extension to flexion without removing the subject from the scanner for re-positioning. The flexed knee was imaged along the same para-sagittal plane as the extended knee. Supports helped the subject maintain a given flexion angle during the MR image acquisition.

All flexion angles were measured using a goniometer (model #32–4100, Almedic, Canada) placed on the surface of the leg, unless otherwise noted. Goniometers have an inaccuracy of up to 10° [11], which is a potential source of error.

### PCL Measurements using 2D Slice Partitions

**Figure 2:**
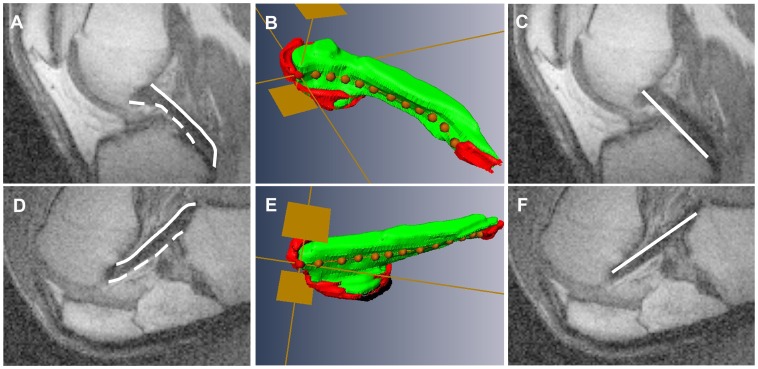
The three measurement methods used. 1. Surface measurements from 2D slice partitions; dashed line on anterior surface, solid line on posterior surface (A, D). 2. Surface measurements from 3D reconstructions. Notice how the PCL bends and twists after flexion. Measurements were taken along the orange dotted line. The red areas are regions where the PCL meets the bone. For clarity, only measurements of the anterior surface of the PCL are shown. (B, E). 3. Straight-line measurements between PCL attachments on 2D slice partitions (C, F).

To simplify terminology in this paper, slices reconstructed from 3D scans along the original para-sagittal imaging plane are referred to as “2D slice partitions”. The PCL appeared in at least three consecutive 2D slice partitions. The middle slice was selected to avoid partial volume artifacts and to measure the PCL’s length as close to the mid-line of the main substance as possible. The selected 2D slice partition was imported into *ImageJ* software (National Institutes of Health, USA). The length of anterior surface of each PCL (e.g. Figure 2A and 2D, dashed line) was measured three times using the segmented line tool in *ImageJ*, and the average of the three measurements was taken as the value for that subject. The same was done for the posterior surface (e.g. Figure 2A and 2D, solid line). None of the data were tested for normality since Gaussian distribution tests rarely detect non-normality when *n*≤40 [23]. However, *t*-tests are robust to moderate violations in assumptions of normality [24], and were used to analysis data in Experiments 1 and 3.

### Experiment 1 – Extension vs. Deep flexion

Eight subjects were recruited. The PCL was clear in the images obtained from 7 out of 8 subjects. The 8^th^ patient’s PCL was not clear, probably due to incorrect placement of the subject in the scanner. Data from this subject were not used. The mean age of the 7 remaining subjects (4 males and 3 females) was 46.1 years (range: 36.5–64.6 years). Mean knee flexion (±S.D.) was 12°±4° in relaxed extension (*n* = 7). Knees were not at 0° for reasons described in “PCL imaging procedures common to all Experiments”. After obtaining images of relaxed extension, knees were flexed to a comfortable maximum indicated by the subject. The mean knee flexion (±S.D.) measured with the goniometer at this maximum was 118°±6° (*n* = 7). Several subjects noted that the RF coil physically prevented deeper flexion angles.

Images used for measurements were reconstructed with 2x zero-filling in all three dimensions using *Marevisi* software (National Research Council of Canada, Canada). The lengths of the anterior and posterior surfaces of the PCL were measured from 2D slice partitions as described in “PCL measurements using 2D slice partitions”. This was performed for extended (Figure 2A) and flexed (Figure 2D) knees for all subjects. Measurements from extended and flexed knees were compared using a two-tailed paired *t*-test.

To ensure that differences between extension and flexion were not due to the PCL moving relative to the imaging plane, the PCL was also measured using 3D reconstructions as follows. The PCL was manually segmented from 2D slice partitions which had been extracted from 3D MRI volumes. The PCL was reconstructed in 3D using *Amira* software (Visage Imaging, Australia). The spline probe was used to measure the surface of the PCL in extended (Figure 2B) and flexed knees (Figure 2E). This was done for both the anterior surface (Figure 2B and 2E) and the posterior surface (not shown). The 3D reconstructions allowed the mid-line of the PCL to be measured in all cases, even if the PCL moved relative to the imaging plane.

Measurements from 2D slice partitions and from 3D reconstructions were compared using two-tailed paired *t*-tests. Also, using 3D reconstructions, the length of the PCL at extension was compared to its length at flexion using a two-tailed paired *t*-test. This was done for anterior and posterior sides to see if conclusions were the same as for 2D slice partitions.

### Experiment 2 – 2D and 3D Videos

To animate the changes seen during knee flexion, two videos were made, each from a different, healthy subject. The first subject was imaged at multiple unloaded flexion angles using the 2D SSFP-FID sequence and parameters listed for Experiment 2 (Table 1). *iMovie* (Apple Inc., USA) was used to create a 2D video of knee flexion from the 2D MR images. The second subject’s unloaded knee was imaged at relaxed extension, and at flexion angles of 40°, 70°, 100° and 120° using the 3D SSFP-FID pulse sequence from Experiment 1 (Table 1). The bones, PCL, cartilage, ALC and menisci were manually segmented from the resulting images. Because the images were acquired using the 3D pulse sequence, tissues were able to be reconstructed in 3D using *Amira* software. *iMovie* was used to create a 3D video of knee flexion from the reconstructions.

### Experiment 3 – Patterns of PCL Lengthening during Flexion

Seven subjects were recruited. Two of these subjects were from Experiment 1 and 5 were new recruits. The mean age of the 7 subjects (4 males and 3 females) was 29.8 years (range: 18.3–64.6 years). A 3D sequence was used for this Experiment to ensure that when the PCL moved laterally during knee flexion, it would still be captured within the imaging volume (Table 1 – Experiment 3). Subjects were imaged in unloaded relaxed extension (13.7°±7.8° for this group). Knees were not at 0° flexion during relaxed extension for the reasons described in “PCL imaging procedures common to all Experiments”. After being imaged in relaxed extension, subjects’ unloaded knees were imaged at 40°, 70°, 100° and 120° flexion (*n* = 7). Five of the subjects were imaged at the additional flexion angles of 90° and 100°.

Images were reconstructed with 4x zero-filling in x and y dimensions using *Marevisi* software. As described below in “Results”, Experiment 1 demonstrated that measurements taken from 2D slice partitions and from 3D reconstructions yielded similar results. 2D slice partitions were quicker to extract and to measure than 3D reconstructions. Therefore, although a 3D volume was acquired to ensure that the PCL was imaged, only one 2D slice partition per subject and flexion angle was selected for measurement. 2D slice partitions were selected as described in “PCL measurements using 2D slice partitions” and imported into *ImageJ*. The anterior and posterior surfaces of the PCL were measured at each flexion angle for each subject using the segmented line tool (e.g. Figure 2A and 2D). Additionally, the straight-line distance between PCL attachments was measured using the straight line tool (e.g. Figure 2C and 2F). Lengths were expressed as % maximum value for that subject and aspect measured. Pair-wise comparisons of length between selected flexion angles were done using one-tailed paired *t*-tests. The Bonferroni Correction was applied when multiple comparisons were made [24].

Five knee flexion angles were collected for all seven subjects in Experiment 3: relaxed extension, 40°, 70°, 100° and 120°. Patterns of increase and decrease over these flexion angles were tested for consistency between subjects using the “Approximate test for trends and contrasts” [25]. This test examines whether the data obtained from each subject follows the same pattern of increases and decreases as the other subjects. The “exact” version of this test is used only when *n*≤4 [25]. Significance was set at 0.05 for all tests.

## Results

### Experiment 1 – Extension vs. Deep Flexion

The anterior surface of the PCL lengthened significantly after unloaded knee flexion (*p*<0.001) according to 2D slice partition measurements (Table 2). Conversely, the posterior surface of the PCL did not change length significantly when the knee flexed (*p* = 0.28).

**Table 2:** The length of the PCL measured from 2D slice partitions and 3D reconstructions.

Surface - type of flexion	2D slice partition (mean±S.D.)	3D reconstruction (mean±S.D.)	*p*-value for 2D–3D comparison
Anterior surface - Extended	32.8±6.8 mm	32.6±6.7 mm	0.91
Anterior surface – Flexed	41.9±4.7 mm*	40.8±7.3 mm*	0.58
Posterior surface - Extended	41.3±6.6 mm	40.1±5.6 mm	0.49
Posterior surface – Flexed	43.0±7.2 mm+	41.5±5.7 mm+	0.13

* *p*<0.001 compared to “Anterior surface – Extended” of same measurement technique.

+ Not significantly different from “Posterior surface – Extended” of same measurement technique (*p* = 0.28 for 2D slice partition comparison; *p* = 0.19 for 3D reconstruction comparison).

To ensure that the above observations were not due to the PCL moving relative to the imaging plane, PCL’s were measured in 3 dimensions. Manual segmentation and 3D reconstruction of the PCL using *Amira* software was possible for all 7 PCL’s, both in extension and in flexion. None of the measurements obtained from 3D reconstructions differed significantly from 2D slice partition measurements (Table 2). Like measurements from 2D slice partitions, measurements from 3D reconstructions also showed that the anterior surface of the PCL was shorter before flexion than after flexion (*p*<0.001), while the posterior surface did not change length (*p* = 0.19, Table 2).

### Experiment 2 – 2D and 3D Videos

**Figure 3:**
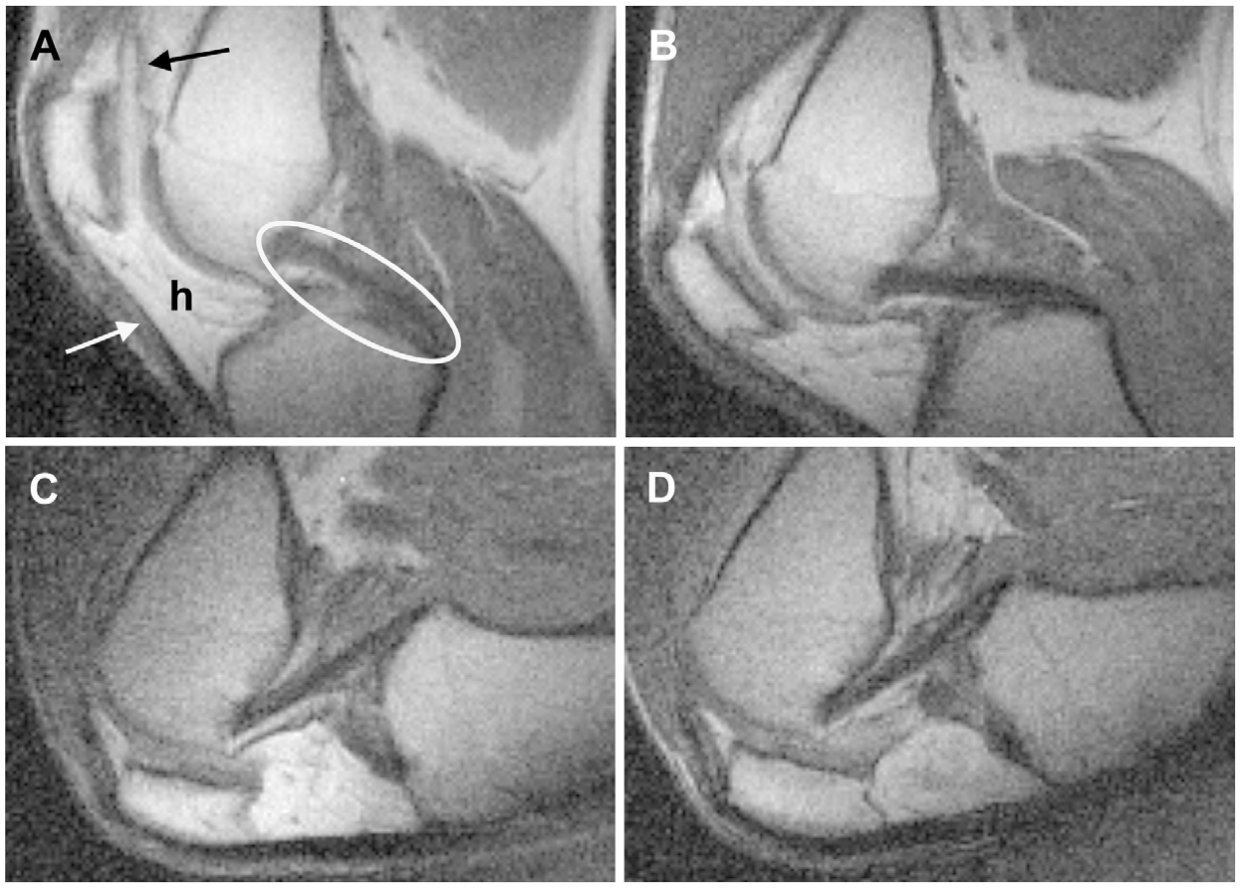
Four of the 2D MR images that were stitched together to make the 2D movie. The anterior of the knee is to the left, the posterior is to the right. Knee flexion angles were approximately 35° (A), 65° (B), 105° (C) and 120° (D). Soft tissues visible include the PCL (oval), patellar tendon (white arrow), Hoffa’s fat pad (h), and synovium (black arrow).

Videos were made successfully from both 2D and 3D MRI pulse sequences. The knee flexion video made from 2D acquisitions provided good visualization of knee soft tissues such as the PCL, patellar tendon, Hoffa’s fat pad and the synovium (Figure 3). The video animated patellar tracking and synovial membrane movement during unloaded knee flexion, highlighting changes that were not obvious from static 2D slices (Video S1). Caution was required when interpreting the 2D video because some soft tissue shape changes were due to the imaging plane moving relative to the soft tissues rather than being due to knee flexion alone; 7 out of 16 acquisitions were too lateral or medial to include the PCL.

**Figure 4:**
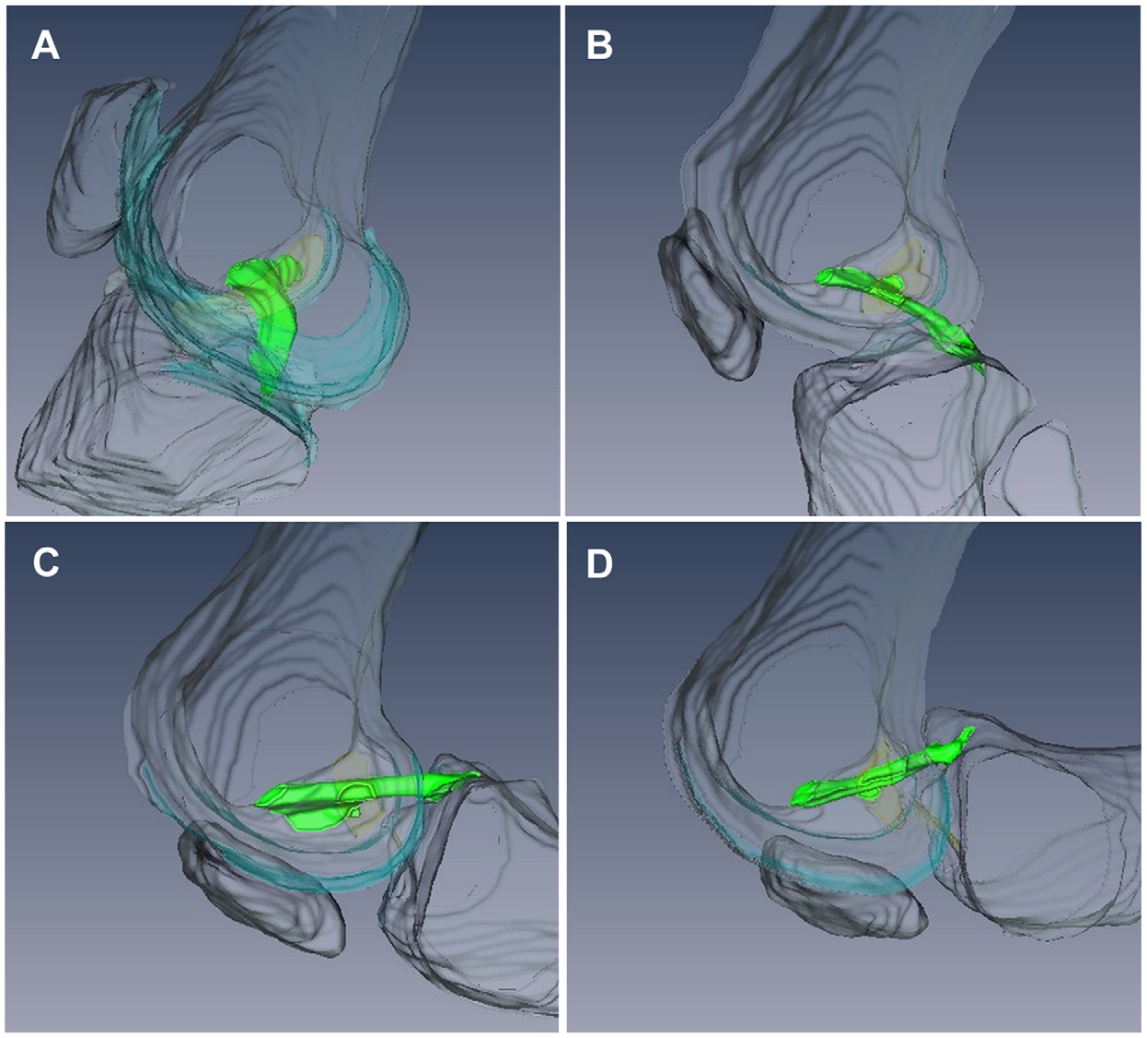
Four of the 3D reconstructions of the bones and PCL that were stitched together to make the 3D movie. Orientation is as in Figure 3. Bones are grey and transparent in order to better view the green PCL. Knee flexion angles were approximately 10° (A), 40° (B), 100° (C) and 120° (D). Notice how the PCL becomes straight at 40° flexion. Transparent blue occurs where the bone meets cartilage. Transparent yellow occurs where the bone meets the anterior cruciate ligament.

For the 3D knee flexion video, the bones, PCL, ACL, cartilage and menisci were manually segmented. However, since the ACL, cartilage and menisci were not the focus of the imaging, and only parts of these tissues appeared in the MR images, they could not be reconstructed in their entirety. They were excluded from the video except to note where the cartilage and ACL touched the bone (Figure 4). However, with a different imaging plane and field of view, these tissues could be reconstructed fully. The video made from 3D reconstructions was more robust to movement of the imaging plane; 5/5 acquisitions included the whole PCL. The complex 3D stretching and bending of the PCL during unloaded flexion could be observed (Video S2). The change in appearance in the PCL between extension, when the PCL was quite curved, and 40° flexion, when the PCL was quite straight, was particularly striking. The limitations of 3D video were that is was more time consuming to produce than the 2D video, and was limited to tissues that were easy to segment (e.g. bones, PCL, menisci).

### Experiment 3 – Patterns of PCL Lengthening during Flexion

**Figure 5:**
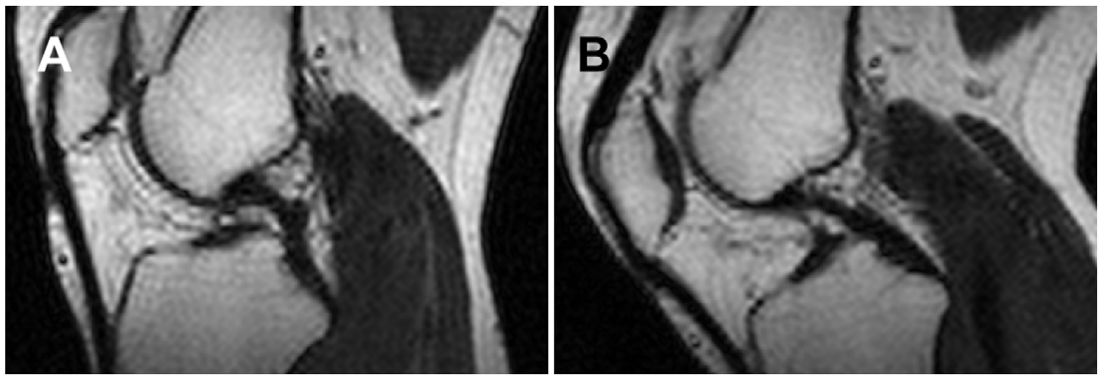
Example images from Experiment 3. The PCL appears curved at relaxed extension (∼16°; A), but relatively straight at 40° (B). The images were windowed for maximum contrast between the PCL and surrounding tissues. Knee orientation is the same as in Figures 3 and 4.

The PCL appeared curved when the knee was in unloaded relaxed extension in 5/7 subjects (Figure 5A). The two subjects in which the PCL was not curved at relaxed extension had their knees at 28° and 30° flexion (measured post-hoc with a goniometer from the bones in the MR images). The 5 subjects with a curved PCL at relaxed extension had their knees at 5°, 7°, 9°, 11° and 16° flexion (measured post-hoc with a goniometer from the bones in the MR images). In 7/7 images taken at 40° flexion, the PCL appeared straight except for what was necessary to bend over the spine of the tibia (Figure 5B).

**Figure 6:**
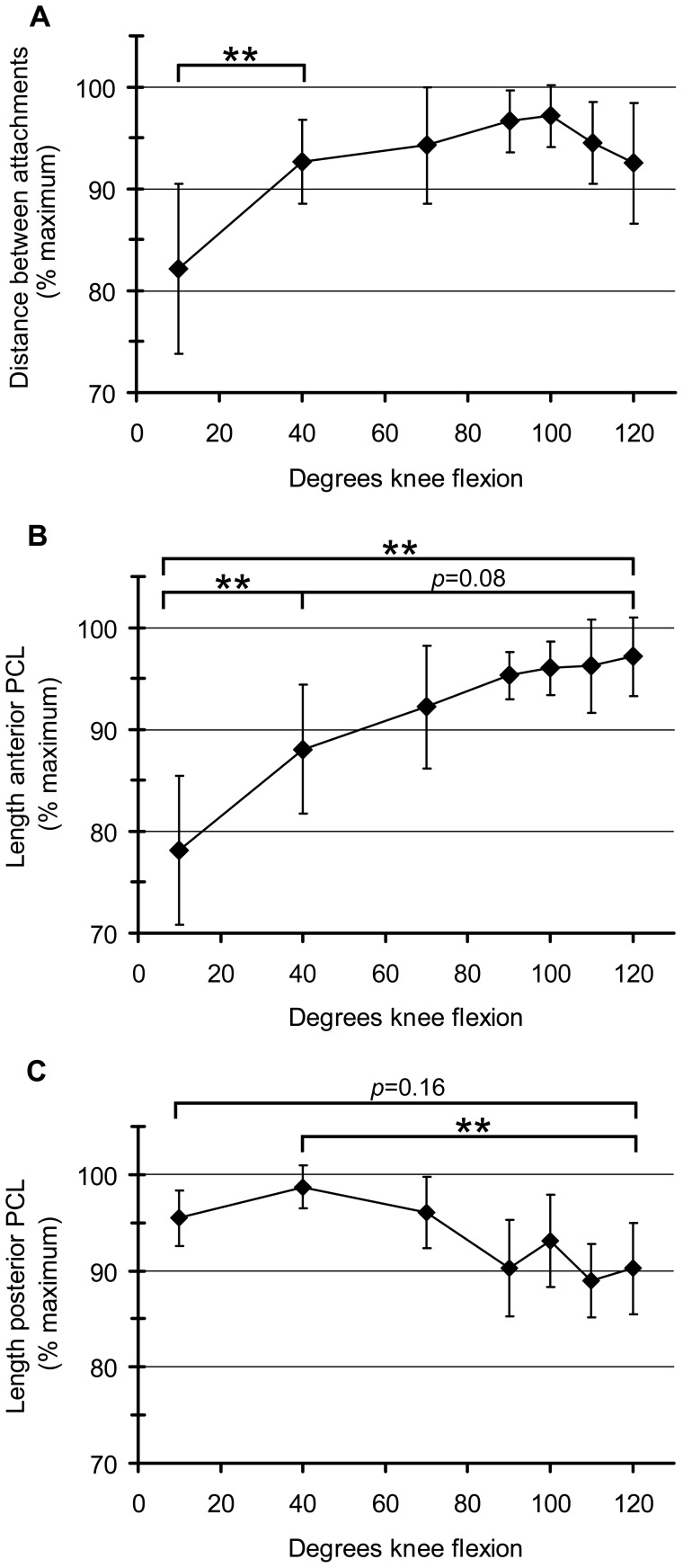
Distance between PCL attachments, and PCL surface length, at flexion angles between relaxed extension and 120 ° **flexion.** Distance between PCL attachments (mean±S.D.) (A). There was a statistically significant pattern of attachment separation as the knee flexed to 100°. Beyond 100° flexion attachments became closer again (*p*<0.001). Length of the anterior surface of the PCL (mean±S.D.) (B). There was a statistically significant pattern of lengthening over the flexion arc to 120° (*p*<0.01). Length of the posterior surface of the PCL (mean±S.D.) (C). There was a statistically significant pattern of lengthening to 40°, then shortening to 120° (*p*<0.001). *n* = 7 for relaxed extension, 40°, 70°, 100° and 120° flexion. *n* = 5 for 90° and 110° flexion. **p<0.01 (paired *t*-tests).

The attachments of the PCL had a statistically significant pattern of separating as the unloaded knee flexed to 100°, and then becoming closer again beyond 100° flexion (*p*<0.001; Figure 6A). There was a large and statistically significant increase in mean attachment separation when the knee flexed from relaxed extension to 40° flexion (10.5%, *p*<0.01). Subsequent intervals showed smaller changes (Figure 6A).

The anterior surface of the PCL had a statistically significant pattern of lengthening over the flexion arc to 120° (*p*<0.01; Figure 6B). The mean length of the anterior surface of the PCL increased significantly in the interval between relaxed extension and 40° flexion (10%; *p*<0.01). Increases in subsequent intervals were smaller, but resulted in an almost significant increase in length between 40° and 120° (9% over combined intervals; *p* = 0.08 after Bonferroni Correction). Like in Experiment 1, the anterior surface of the PCL was significantly longer at 120° than at relaxed extension (*p*<0.01).

The posterior side of the PCL had a statistically significant pattern of lengthening between relaxed extension and 40°, and then shortening again between 40° and 120° (*p*<0.001; Figure 6C). The posterior surface of the PCL was significantly shorter at 120° than at 40° (9%, *p*<0.01). However, like in Experiment 1, the length of the posterior side of the PCL was not significantly different between relaxed extension and 120° flexion (*p* = 0.16).

## Discussion

By directly measuring the length of the PCL in an open-bore MRI, we were able to detect statistically significant patterns in PCL deformation over the flexion arc from relaxed extension (i.e. extension existing in a subject lying on their side, muscles relaxed) to 120° flexion, in the unloaded knee. This arc included about 75° of flexion that cannot be investigated by most closed-bore MRI scanners, and included full coverage of the “active function” sub-arc in which most daily activities occur [11]. Each of the three PCL parameters measured (distance between attachments, length of anterior side, and length of posterior side) lengthened between relaxed extension and 40°, but showed different trends beyond 40° flexion. This indicates that the PCL deformed differently between the terminal extension and active function sub-arcs. Therefore, not only are bone biomechanics different between these sub-arcs, but PCL deformation is as well. The distance between PCL attachments followed the same pattern of increasing and decreasing that has been found previously in unloaded flexing knees *in vivo* [14]. The lengths of the anterior and posterior surfaces of the PCL have not been measured previously *in vivo*. We found that above 40° flexion, each of the three PCL parameters measured (distance between attachments, length of anterior side, and length of posterior side) showed different patterns of lengthening and shortening, indicating that deformation of the surfaces of the PCL cannot be inferred from PCL attachment separation. Likewise, deformation of one surface of the PCL cannot be used to infer deformation of another. The deformation of a given region of the PCL needs to be measured directly, at the flexion angles of interest.

One similarity that all three PCL measurements (distance between attachments, length of anterior side, and length of posterior side) displayed was an increase between extension and 40°. It is possible this was due to slack being taken out of at least some parts of the PCL. Studies have reported that the PCL appears slack when the knee is extended, but taut at higher flexion angles [12], [14]. The appearance of the PCL in the 3D video (Figure 4) and in Experiment 2 (Figure 5) indicated that, in our study, this change in appearance occurred between relaxed extension and 30°–40° flexion in the unloaded knee. The hypothesis that slack is being taken out of the PCL between relaxed extension and 30°–40° flexion is consistent with the finding that structures other than the PCL are primarily responsible for knee stability between 0° and 30° flexion in cadaveric knees [26], [27], [28]. It is also consistent with the finding that supine PCL-deficient patients experience more knee instability at flexion angles above 30° [29].

It should be noted that knee bone kinematics are activity-specific [30], [31], and could differ between supine and standing versions of the same activity [29], [32]. This difference in bone kinematics is probably due to differing muscle recruitment between activities. Activity-specific bone kinematics likely result in activity-specific PCL deformation; the PCL deformation we measured during open kinetic chain movement should not be assumed to apply to all activities. The differences in PCL deformation between different activities could be investigated using techniques similar to those presented here. Open-bore scanners can be built with a vertical bore [32], which allows a subject to stand, squat and perform everyday activities such as running or stair climbing. As technology improves, images of the joint soft tissues could be acquired during dynamic activity and measured for deformation. Electromyogram leads could be used simultaneously to measure muscle activation, elucidating the connection between muscle contraction, bone kinematics and PCL lengthening or other PCL deformation.

The lengthening of the PCL surfaces observed in our study could be associated with loading in the following way. It may be that the whole ligament is loaded at 40°, but that the posterior surface of the PCL is relatively more loaded than other parts. The posterior surface would therefore lengthen, due to strain. As the knee flexes beyond 40°, and the orientation of the PCL attachments change relative to each other, load may be taken off the posterior surface which would subsequently shorten due to viscoelasticity (at 120° the posterior surface is 9% shorter than its maximum length in this study at ∼40°). Simultaneously, the load would be moved anteriorly through the substance of the ligament. Loaded regions would lengthen due to strain and then shorten when they became unloaded at higher flexion angles. The load would reach and strain the anterior surface at about 120° causing it to lengthen from its just taut length at 40° flexion (9% lengthening observed in our study between 40° and 120°). This model is an oversimplification of PCL anatomy; for example, the anterior and posterior PCL surfaces measured in this study did not correspond anatomically to any bundles identified within the PCL [33]. However, our model supports hypotheses [34], [35], [36] that there are at least two regions within the PCL, and possibly more, that function differently over the knee flexion arc. Although we do not know whether the PCL was loaded as described above, our data suggest that the PCL contributes to knee stability. The anterior surface of the PCL lengthened by a surprising 24% between extension and flexion (values from Experiment 1). It would be expected to start bearing load at some point during this lengthening. If the anterior surface of the PCL is loaded, it must be restraining the relative motion of the tibia and femur, i.e. it must be contributing to joint stability.

To truly test hypotheses about whether parts of a ligament are slack, just taut or loaded at different flexion angles *in vivo*, it will be necessary to identify MRI qualities that are visible in open-bore scanners and that indicate ligament loading. For example, high-field (9.4T) closed-bore MRI found that “crimp”, which is visible in MR images, decreased in loaded tendons, while the MRI relaxation parameter “T_2_” simultaneously increased [37]. Further testing is required to determine whether the same qualities can be used to identify loading in the cruciate ligaments, which have structural differences from tendons [38]. By combining load information with lengthening data, the biomechanics of soft tissues in the flexing knee could be elucidated.

### Twisting of the PCL Relative to the Imaging Plane

Imaging, and therefore measuring, the exact same part of the PCL over the flexion arc is complicated. The PCL twists and bends during flexion, causing some parts to remain in the imaging plane, and others to move out. To test the extent of this potential error, we used two measurement techniques. In the first, we measured lengths in a 2D slice partition, as has been done previously [14], [15]. In the second, we reconstructed the PCL in 3D. PCL length was then measured along the PCL’s midline, even if this meant measuring across imaging planes (Experiment 1). Measurements made from 3D reconstructions were similar to those made from 2D para-sagittal slice partitions (Table 2). This indicates that the twisting and bending of the PCL relative to the imaging plane was not responsible for the length changes observed in Experiments 1 and 3.

### Previous Studies

One other study has measured the length of the posterior surface of the PCL at multiple unloaded knee flexion angles between extension and 120° flexion [14]. That study used cadaver knees and did not find that the posterior surface of the PCL changed length. Conversely, we found that the PCL was significantly longer at 40° than at 120° flexion. Three reasons might explain why the results differ. First, instead of measuring the posterior surface of the PCL directly, the previous investigators fitted a strip of malleable modeling material to the posterior border of the PCL. The modeling material was then straightened for measurement. It is unclear how feasible it would be to straighten the modeling material without changing its length, or where across the thickness of the modeling material the measurements were taken. Both could have affected measurement accuracy. A second possible reason for the discrepancy between their results and ours is that the mean distance between PCL attachments was significantly larger in their cadavers than in their living subjects at extension and at 120° flexion. The reason for this difference is unknown, but indicates that knee geometry in the cadavers was different than in the live subjects. This difference in geometry could affect deformation of the posterior surface of the PCL during flexion. Third, another study has shown that the extension/flexion path of the bones in a cadaveric knee is variable and differs from the bone path observed *in vivo* [39]. Although movement in our study was “relaxed”, the baseline muscle activity of a living person could have resulted in bone positions that were different from those existing in cadavers. Different bone positions would likely result in different PCL deformation. No other study has measured the length of the anterior or posterior side of the PCL during flexion *in vivo*.

### Knee Videos

The 3D reconstructions were helpful for understanding the overall anatomy of the knee. Because of their utility, 3D renderings are also used in commercially available artists’ renditions of knee ligament deformation such as those from Anatomy.TV (Primal Pictures, United Kingdom). To capitalize on the potential of multiple MR images to show the way actual soft tissues move relative to each other, we made a video which stitched several acquisitions together. There are no previous examples of videos of the soft tissues inside the *in vivo* human knee during knee flexion, unloaded or otherwise. Videos could be made of the knee bones and surrounding muscles from images obtained from cine-PC MRI and real-time MRI [40] to facilitate visualization of their movement during joint flexion.

Because the PCL was the focus of our study, the slice in which the PCL occurred was chosen for our 2D videos. However, other tissues were also visible, especially in the 3D images, including the synovium, patella, patellar tendon, ACL, Hoffa’s fat pad, cartilage, menisci and bones. The relationship between the movement and deformation of the different tissues could be easily observed. 3D videos provided the added benefit of capturing tissues even when they moved relative to the imaging plane. Videos of the flexing knee could provide an important tool for teaching and understanding integrated joint biomechanics.

### Conclusions

The current study demonstrates the considerable potential of open-bore scanners to investigate soft tissues of the knee over the entire active function sub-arc. The distance between PCL attachments, the length of the anterior surface of the PCL and the length of the posterior surface of the PCL all changed in a consistent, but different pattern over the flexion arc between unloaded relaxed extension and 120°. The PCL’s course relative to the femur changes throughout flexion. Measurements made from 3D models that compensated for this did not differ significantly from measurements made from 2D para-sagittal slices. Therefore, the PCL’s changing course during flexion was not the cause of the length changes observed here. Data supported hypotheses that parts of the PCL are slack over the terminal extension sub-arc and that the PCL is not uniformly loaded over the active function sub-arc during open kinetic chain movement. Data indicated that the deformation of one part of the PCL cannot be inferred from the deformation of another part, nor can it be inferred from attachment separation in the unloaded knee. Data also indicated that the PCL deformed differently from one flexion sub-arc to the next. Therefore, the biomechanics of the PCL in any given region and for any given sub-arc should be measured directly in order to understand the PCL’s role in knee stability.

## Supporting Information

Video S1 **Video made from static 2D MR images that were stitched together.** Images are para-sagittal with the anterior of the knee to the left and the posterior to the right. The video provides good visualization of many soft tissues, including the PCL, the patellar tendon, Hoffa’s fat pad and the synovium (see Figure 3).
(MP4)

Video S2 **Video made from five static 3D MR acquisitions that were reconstructed in 3D and stitched together.** The bones are in grey and the PCL is in green. The initial rotations of the extended knee demonstrate how difficult the PCL is to access anteriorly. After the initial rotations, the bones become transparent to reveal shape changes of the PCL during knee flexion. PCL is viewed from the lateral side. The 3D video is more robust to lateral movement of the imaging plane than the 2D video (Video S1). As in Figure 4, transparent blue occurs where bone meets cartilage; transparent yellow occurs where bone meets the anterior cruciate ligament.
(MP4)
